# Letter to the editor: prediction of hematogenous metastasis risk from molecular classification in gastric cancer

**DOI:** 10.1097/JS9.0000000000003316

**Published:** 2025-08-26

**Authors:** Wei Zhao, Wenqing Bao, Ning Wu

**Affiliations:** Department of Oncology, Gongli Hospital of Shanghai Pudong New Area, Shanghai, China

*Dear Editor,*

We read with great interest the recently published article entitled *“Prediction of hematogenous metastasis risk from molecular classification in gastric cancer”*^[1]^. This study identified molecular subtypes associated with hematogenous metastasis in gastric cancer through integrative multi-omics analysis and established multiple machine learning models to predict the risk of hematogenous spread. While the work provides valuable insights into the mechanisms of distant metastasis and holds potential for clinical application, we believe several important aspects merit further discussion. This article was written under the guidance of TITAN^[2]^.

The authors employed non-negative matrix factorization (NMF) clustering on bulk RNA-seq data from 64 gastric cancer samples to define molecular subtypes. We suggest that the robustness of this classification could be further supported by comparing results from additional unsupervised clustering methods, such as consensus clustering or hierarchical clustering^[3]^. Assessing the consistency across methods may help confirm the stability of subtype assignment. Furthermore, in estimating tumor microenvironment composition, future analyses could benefit from the use of the *immunedeconv* R package^[4]^, which integrates multiple algorithms – including quanTIseq, TIMER, CIBERSORT, CIBERSORT-ABS, MCP-counter, xCell, EPIC, ABIS, ConsensusTME, and ESTIMATE – for comprehensive immune cell deconvolution and expanded profiling of immune components.

While multiple machine learning approaches were evaluated and a gradient boosting survival model was ultimately selected, the model performance was primarily assessed using the ROC curve and AUC values. However, key metrics such as the confusion matrix, sensitivity, and specificity were not reported, making it difficult to assess the model’s practical utility in a clinical context. Incorporating interpretability methods such as SHAP could also provide insights into gene-level contributions and improve transparency.

The definition and labeling of hematogenous metastasis (HM) require further clarification. The study refers to HM cases as patients with “evidence of distant or hematogenous metastasis,” yet it is unclear whether this determination was based on imaging, pathology, or follow-up data. Particularly in public datasets like TCGA and GEO, detailed records of metastatic status are often lacking, potentially introducing label noise and affecting the quality of model training. Including sequencing data from the authors’ own institutional cohort could help address this issue.

Although the study explores immune and molecular features of the HM subtype, the absence of functional validation, such as qPCR, immunohistochemistry, or pathway-level assays, limits the biological interpretation of key molecules or mechanisms involved in hematogenous dissemination. Experimental verification, for example using CRISPR or RNAi knockdown in PDX or organoid models, would substantially strengthen the mechanistic claims.

Finally, the clinical applicability of the model remains uncertain. Despite its predictive performance, the model has not been tested in real-world prospective clinical cohorts, nor has its impact on treatment decision-making or survival outcomes been evaluated. Moreover, the use of a 17-gene panel for prediction may raise concerns regarding cost-effectiveness and accessibility in routine practice.

In summary, we commend the authors for their innovative efforts to predict hematogenous metastasis in gastric cancer. To enhance scientific rigor and translational value, we encourage further validation of subtype reproducibility, refinement of model-building strategies, clarification of metastasis definitions, incorporation of biological experiments, and assessment of clinical impact.

## Data Availability

Not applicable.
